# The expression of the GATA6 gene in oral carcinoma cell lines

**DOI:** 10.1186/s12957-021-02245-y

**Published:** 2021-05-18

**Authors:** Cheng-Lin Xu, Wei-Qun Guan, Xue-Ying Wang

**Affiliations:** 1 Department of Stomatology, Affiliated Hospital of Putian University, Putian, 351100 China; 2 Department of Stomatology, Union Hospital, Fujian Medical University, No. 29 of Xinquan Street, Gulou District, Fuzhou, 350001 China

**Keywords:** GATA6, Oral cancer, RT-PCR, Western blot, Oral epithelial cell

## Abstract

**Background:**

This study aimed to investigate the expression level of the GATA6 gene in different oral cancer cells.

**Methods:**

In this study, we sub-cultured normal oral epithelial cell lines HOK, human tongue squamous cell carcinoma cell lines CAL-27 and SCC-4, and human salivary gland adenoid cystic carcinoma cell lines SACC-LM and SACC-83. Subsequently, we used reverse transcription-polymerase chain reaction RT-PCR and Western blot methods to detect the mRNA and the protein expressions of GATA6 in normal oral epithelial cells, human tongue squamous cell carcinoma cells, and human salivary gland adenoid cystic carcinoma cells.

**Results:**

The results of this study showed that the mRNA expression levels of GATA6 in CAL-27, SCC-4, and SACC-LM cells were significantly increased when compared with the HOK cells. However, the mRNA expression level of GATA6 in the SACC-83 cells had no significant difference compared with the HOK cells. The protein expression levels of GATA6 in the SCC-4 and SACC-LM cells were, however, significantly increased whereas the protein expression levels of GATA6 in the CAL-27 and SACC-83 cells had no significant difference when compared with the HOK cells.

**Conclusion:**

The GATA6 gene may be related to the occurrence and progression of certain oral cancers.

## Background

Oral cancer is one of the most frequent malignancies of the head and neck. Recently, the incidence of certain oral cancer has increased yearly, with strong local infiltration and cervical lymph node metastasis as the main characteristics of oral cancer in patients. However, the 5-year survival rates for oral cancer are still low [1–4]. Recent studies have reported that GATA6 has a basic expression in a variety of tumors, the levels of expression vary significantly depending on the tissue source, and it plays different regulatory roles in the process of tumor development [5, 6].

Consequently, we conducted this study to examine the expression level of the GATA6 gene in oral cancer cells.

## Methods

### The experimental cell lines

In this study, the human tongue squamous cell carcinoma cell lines SCC-4, human salivary gland adenoid cystic cell lines SACC-LM and SACC-83, and the human normal epithelial cell lines HOK were purchased from Shanghai Yaki Biotechnology Co. Ltd. The human tongue squamous cell carcinoma cell lines CAL-27 were donated by Professor Lu Youguang of the Affiliated Stomatological Hospital of Fujian Medical University.

### The cell cultures and serial sub-cultivation

We first took the cells out from the liquid nitrogen storage. After the dissolution was completed in a water bath at 37 °C, the cells were centrifuged and re-suspended. The cells were then cultured in a cell dish containing an appropriate amount of culture medium in an incubator at 37 °C and with 5% of CO_2_. When the cell density grew to 80% of the surface of the culture plate, the cells were increased by serial sub-cultivation at a ratio of 1:2. The cells were then digested with 1 mL of trypsin for 3–10 min. When the intercellular space was enlarged, rounded, and partially detached under the microscope, 2–3 mL of a serum-containing medium was added immediately to terminate the digestion. The cell suspension was centrifuged, and the cells were re-suspended in 2–3 mL of a DMEM medium containing 10% of FBS. We then divided the cell suspension into 2–3 Petri dishes and added a suitable amount of culture medium for further cultures.

### The reverse transcription-polymerase chain reaction RT-PCR analyses

Total RNA was extracted through the TRIzol method. After measuring the total RNA concentration of the sample, the corresponding cDNA was synthesized by using RNA as the template through reverse transcription. The cDNA product after the reverse transcription was completed was diluted 10 times. The product was labeled with the SYBR® Green I fluorescent dye. The Applied Biosystems® 7500 real-time PCR System was used for the relative quantitative detection of the target genes, with β-actin used as the internal reference. The quantitative PCR reaction system was as follows: SYBR® Premix Ex Taq™II (×2) 12.5 μL, PCR Forward Primer (10 μM) 0.5 μL, PCR Reverse Primer (10 μM) 0.5 μL, RoxII (×50) 0.5 μL, RT reaction liquid (cDNA as a template) 1.5 μL, ddH_2_O9.5 μL, and total volume: 25 μL. The PCR amplification reaction procedure was as follows: pre degeneration 95 °C, 30 s, PCR reaction, 40 cycles were amplified; 95 °C 5 s, 60 °C 34 s. The PCR primers are shown in Table 1.

**Table 1:** RCR reaction primer sequence

Name of the primer	Primer sequences(5′→3′)
GATA6-1-Fw	CTCAGTTCCTACGCTTCGCAT
GATA6-1-Rv	GTCGAGGTCAGTGAACAGCA
GATA6-2-Fw	CTGCGGGCTCTACAGCAAG
GATA6-2-Rv	GTTGGCACAGGACAATCCAAG
GATA6-3-Fw	GTGCCAACTGTCACACCACA
GATA6-3-Rv	GAGTCCACAAGCATTGCACAC
β-actin-Fw	CATGTACGTTGCTATCCAGGC
β-actin-Rv	CTCCTTAATGTCACGCACGAT

### Western blot

When the growth density of the cells reached 80–90%, the cells were removed from the incubator, digested by trypsin to obtain the cell precipitation, and 1 mL of RIPA Lysate was added to lyse the cells. The protein concentration of the samples was determined by the BCA assay. Sodium dodecyl sulfonate-polyacrylamide gel electrophoresis (SDS-PAGE) was used and the cells were transferred to polyvinylidene fluoride (PVDF), which was slowly shaken with the shaking table, and was then sealed with skim milk at room temperature for 1 h. After the primary and secondary antibody incubation, the PVDF membrane was dried with filter paper and chemical fluorescence was used for exposure development. We then obtained the quantitative information of the strip and exported the images.

### Statistical analysis

We used the software program SPSS 20.0 (IBM, Chicago, USA) and GraphPad Prism 5.0 to conduct the statistical analysis. Each set of results that are shown is demonstrative of at least 3 separate experiments. The continuous variables of normal distribution were expressed as mean ± standard deviation, the continuous variables of non-normal distribution were expressed as median (interquartile range [IQR]), and the categorical variables were expressed as frequency (percentage [%]). For two comparisons, each value was compared by a *t* test when each datum conformed to a normal distribution while the non-normally distributed continuous data were compared using non-parametric tests. The counting data were tested by a chi-square test and a value of *P* < 0.05 was considered statistically significant.

## Results

The outcomes of this study showed that the mRNA expression levels of GATA6 in CAL-27, SCC-4, and SACC-LM cells were significantly increased when compared with the HOK cells. However, the mRNA expression level of GATA6 in the SACC-83 cells had no significant difference when compared with the HOK cells. The protein expression levels of GATA6 in the SCC-4 and SACC-LM cells, however, were significantly increased when compared with the HOK cells. The protein expression levels of GATA6 in the CAL-27 and SACC-83 cells had no significant difference when compared with the HOK cells.

### The growth of the cells

**Fig. 1:**
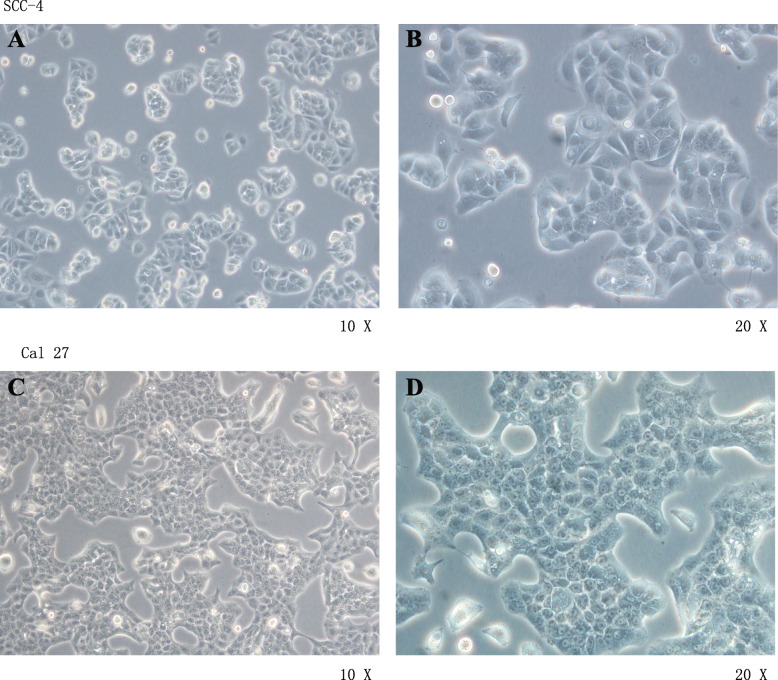
The cells grow. **a** SCC-4 culture day 2 (×10). **b** SCC-4 culture day 2 (×20). **c** CAL-27 culture day 2 (×10). **d** CAL-27 culture day 2 (×20)

Cell lines SCC-4 and CAL-27 were used as examples. The cells grew well in the incubator at 37 °C and with 5% of CO_2_. The outline of the cells was clear, which appeared to be round and oval. The epitheliums presented a monolayer structure, the cells were plump and uniform, the nucleoli were obvious, the cells grew vigorously, and the intercellular structures were closely arranged (Fig. 1).

### The primer quality detection and analysis

**Fig. 2:**
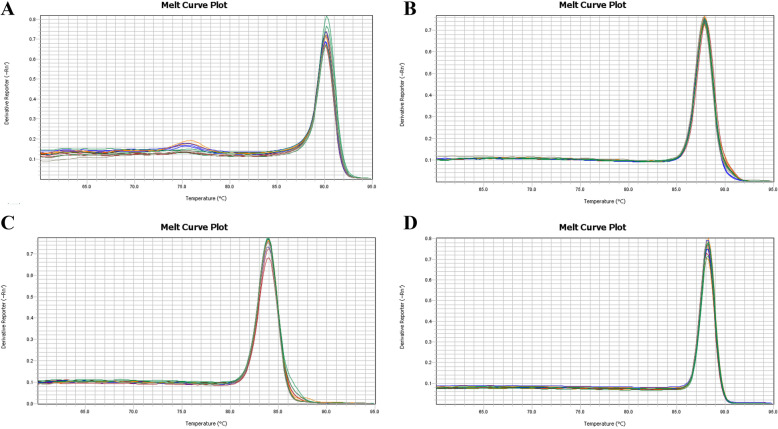
Primer quality detection and analysis. **a** Analysis of primer 1 melting curve. **b** Analysis of primer 2 melting curve. **c** Analysis of primer 3 melting curve. **d** Analysis of primer β-actin melting curve

According to the primer quality test, the quality of primer 1 of GATA6 was not as good as that of primers 2 and 3. The evaluation criteria were as follows: the dissolution temperature was better at 80–90 °C and primer 1 exceeded 90 °C. Actin primers were set as internal parameters. Therefore, primers 2 and 3 were used as PCR primers (Fig. 2).

### Quantitative real-time PCR analysis

All the experimental samples were tested in parallel 3 times and the average values were taken as the final experimental data. The data obtained from the fluorescence quantitative analyzer were examined by a 2^ΔΔCT^ method. These results are listed in Table 2.

**Table 2:** Fluorescence quantitative analysis scale

RQ CT	HOK	SCC-4	SACC-LM	SACC-83	CAL-27
**Primer 1**	1.0000	6.8750	3.7761	0.8837	2.2636
	1.0000	7.5396	3.9009	0.8822	2.2975
	1.0000	7.0804	3.5810	0.9803	2.3805
**Primer 2**	1.0000	6.2455	3.8101	0.8906	2.2449
	1.0000	7.0001	4.2379	1.0161	2.4673
	1.0000	6.6707	3.6934	0.9546	2.2073

**Fig. 3:**
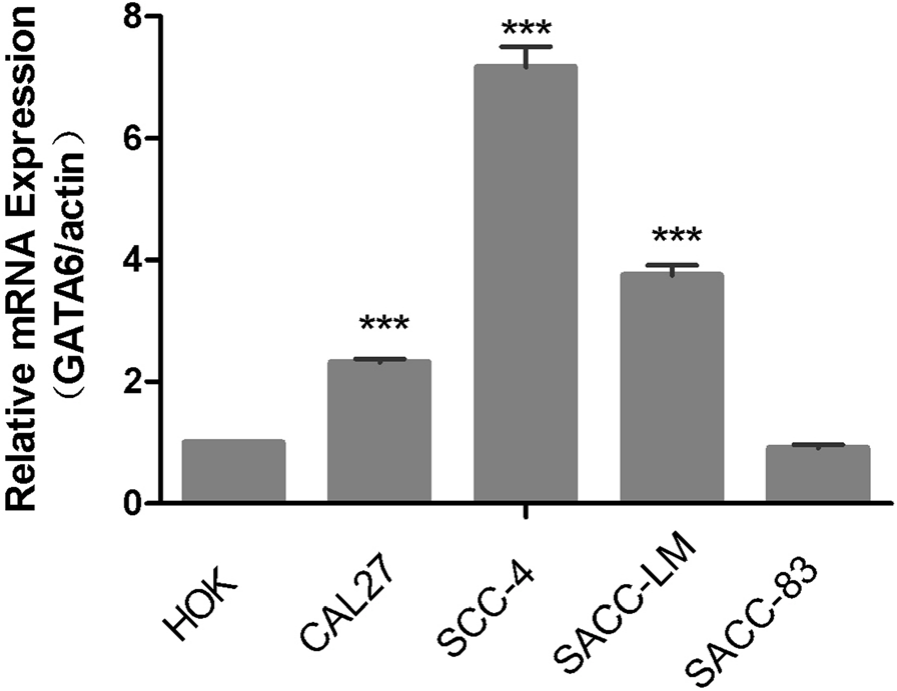
Expression of GATA6 mRNA in human oral squamous cell lines and adenoid cystic cell lines, as shown in the sequence of primer 2

**Fig. 4:**
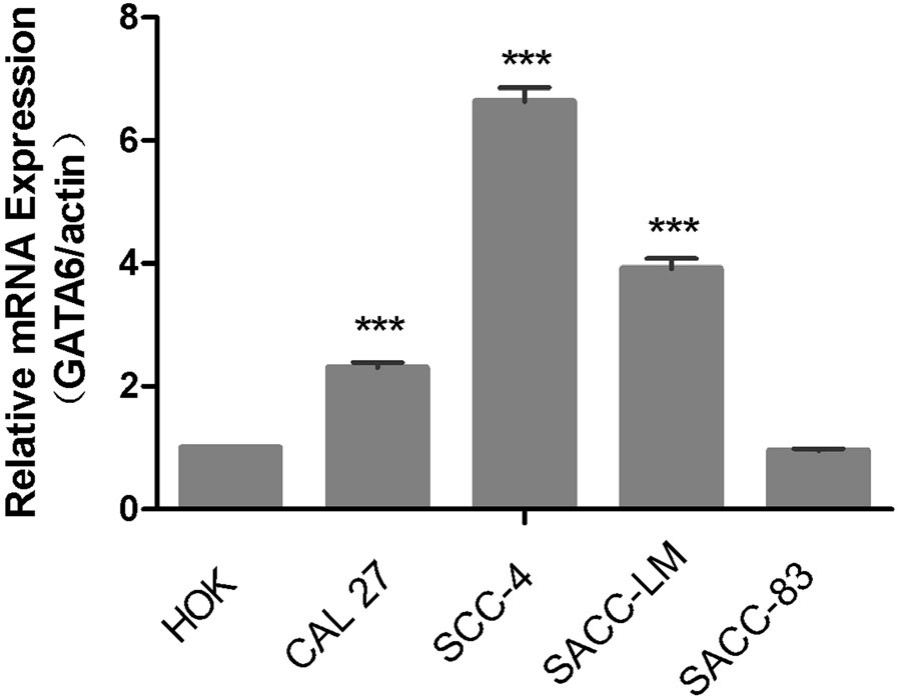
Expression of GATA6 mRNA in human oral squamous cell lines and adenoid cystic cell lines, as shown in the sequence of primer 3

The mRNA expression level of GATA6 was detected by real-time quantitative PCR. We found that compared with the HOK cells, the mRNA expression levels of GATA6 in the CAL-27, SCC-4, and SACC-LM cells were significantly up-regulated. The difference was statistically significant (*P* < 0.05). The mRNA expressions of GATA6 in the SACC-83 cells were not significantly different from those in the HOK cells (*P*  > 0.05) (Figs. 3 and 4).

### The results of the Western blot

**Fig. 5:**
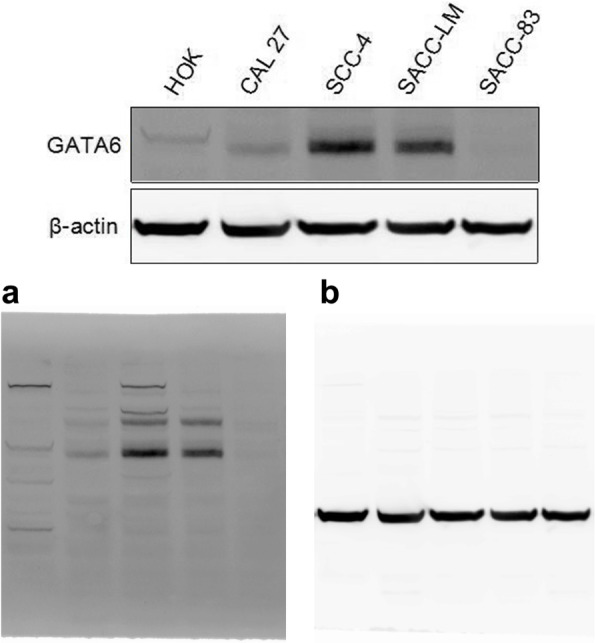
Expression of GATA6 protein in human oral squamous cell linesand adenoid cystic cell lines. **a**, **b** The full, uncropped images of all of Western blots

**Fig. 6:**
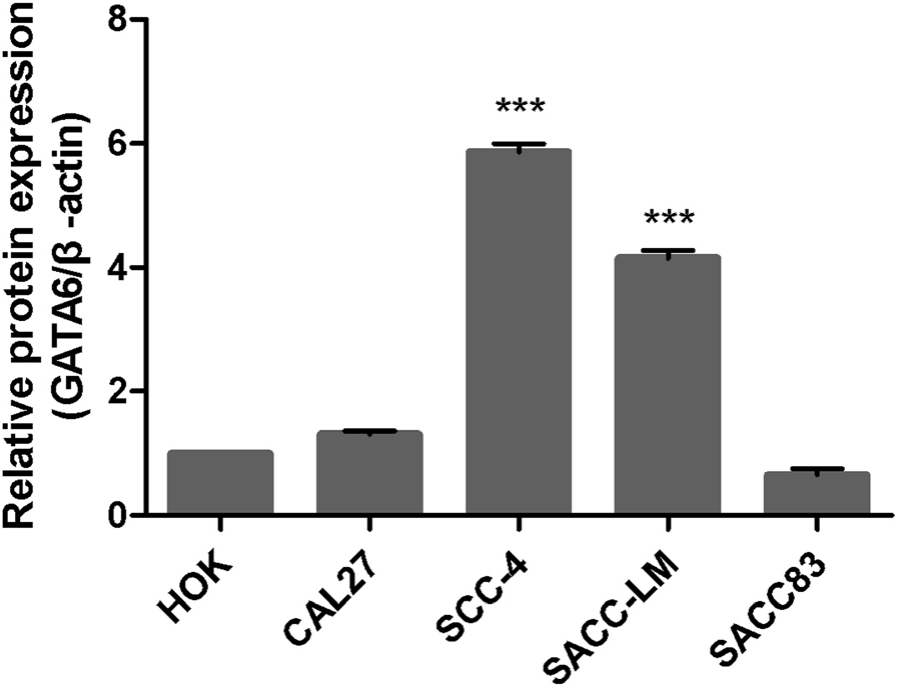
Relative expression of GATA6 protein in human oral squamous cell lines and adenoid cystic cell lines

The Western blot data are shown in Fig. 5. The grayscale analysis of PVDF membrane images of GATA6 protein in each group was performed. The experiment was repeated 3 times and the data of each group are shown in Fig. 6.

## Discussion

GATA6 is a member of the GATA transcription regulator family. As a gene that is closely related to the occurrence and development of diseases, it is involved in a variety of biological behaviors of cells and has a significant relationship with the process of different tumors [7–9]. Therefore, it is of great importance to fully understand and study the GATA6 gene. In a variety of tumor diseases, the role of GATA6 is also complex and varied [10–14]. A study showed that miR-506 can decrease protein levels of GATA6, and GATA6 overexpression reduced the suppressive effects of miR-506. In addition, GATA6 increased in oral carcinoma tissues compared with the adjacent normal tissues [15].

In studying the expression of GATA6 in oral cancer cell lines and understanding its role in the development of oral cancer, research on GATA6 will provide innovative ideas for tumor prevention measures, targeted therapy, and other treatment methods.

Among the four oral cancer cell lines selected in the experiment, GATA6 was expressed in both mRNA and protein levels in two cell lines, only in mRNA level in one cell line and no expression in one cell line. The two cell lines that were expressed were divided into SCC-4 and SACC-LM, and it was speculated that their expression might be independent of the sampling site of the carcinoma tissues to which the cell lines belonged. Previous research has confirmed that GATA6 is expressed in oral squamous cell carcinoma tissues of 21 groups, and the expressions of GATA6 were different due to the different clinical stages, pathological grades, and potential metastatic ability of the selected cancer tissues [15]. Some studies have also found the expression of GATA6 in laryngeal cancer and the corresponding para cancer tissues and normal laryngeal mucosal tissues of healthy people. When compared with normal tissues, GATA6 was highly expressed in laryngeal cancer and para cancer tissues, but the expression of GATA6 in laryngeal cancer tissues was different and was highly linked with lymph node metastasis, clinical staging, and pathological grading [16]. Another researcher used immunohistochemistry to detect the expression of GATA6 in endometrial cancer tissues and the expression difference of GATA6 with atypical endometrial hyperplasia and normal endometrial tissue was compared. Using a data analysis of the relationship between GATA6 and various parameters of endometrial cancer, the findings showed that the expression rates of GATA6 in endometrial cancer tissues, atypical endometrial hyperplasia tissues, and normal endometrial tissues were 63.4%, 18.3%, and 6.0%, respectively. It was also suggested that the distinctive degree of endometrial cancer, TNM stage, distant metastasis, and the infiltration depth of the muscular layer was closely related to the expression of GATA6 [17].

Meanwhile, research based on whether the cell lines can fully represent the primary tumor tissues remains controversial. There have been studies on the comprehensive comparison of copy number variation (CNV), mutation, mRNA expression, and protein expression in breast cancer cell lines and primary breast cancer tissue samples. The findings of these studies showed that the molecular characteristics of breast cancer tumors and cell lines were similar and different. These cell lines reflect only some of the molecular properties of the primary tumor and not all [18]**.** The cancer cells that were grown in vitro also have unstable heredity, which may produce genetic variations during repeated screening, resulting in differences in the expressions [19]. Gene expressions have two levels of transcription and translation and although they are both coupling processes, there is not necessarily a consistent trend. mRNA levels are mainly related to the activation of promoters and enhancers upstream of mRNA, while protein levels are mainly related to function. The gene transcription process to mRNA and then to the proteins requires multiple levels of regulation. It may be transcribed to mRNA and then degraded or it may encounter other signals that stop at the translation level. Gene regulation is not only a transcriptional level as post-transcriptional regulation and post-translational regulation will also have a certain impact on the expression of the subsequent protein levels. Factors such as protein degradation and modified folding may also affect this process. The expression of many genes varies over time, with peaks (high) and valleys (low). It has been reported that protein expression lags behind mRNA by approximately 3 h and the different detection time points may also affect the expression outcomes. These possibilities are worthy of further analyses by selecting more oral cancer cell lines and adjusting the expression time of the varying detected proteins.

This study preliminarily established the expression of the GATA6 gene in oral cancer cells, but there were differences in the protein-level and gene-level expression in the different cell lines, revealing that GATA6 may be involved in the incidence of oral cancer. However, specific mechanisms still need further research.

## Conclusion

This experiment preliminarily confirmed the expression of GATA6 in some oral cancer cell lines. It was positively correlated with the differentiation degree of the cancer tissue and the clinical TNM stage. The overall aim of this study was to examine the role of GATA6 in the development of oral cancer. Further research is still needed to investigate the role of GATA6 in the incidence and progression of oral cancer.

## Data Availability

We declared that materials described in the manuscript, including all relevant raw data, will be freely available to any scientist wishing to use them for non-commercial purposes, without breaching participant confidentiality.

## Abbreviations

SDS-PAGE: Sodium dodecyl sulfonate-polyacrylamide gel electrophoresis
PVDF: Polyvinylidene fluoride
CNV: Copy number variation
